# Distress, Appraisal, and Coping Among the Frontline Healthcare Provider Redeployed to the Epicenter in China During COVID-19 Pandemic

**DOI:** 10.3389/fpsyg.2021.678369

**Published:** 2021-07-30

**Authors:** Zhongliang Ji, Wei Han, Zhe Deng, Kailin Lu

**Affiliations:** ^1^Department of Emergency Medicine, Shenzhen University General Hospital, Shenzhen, China; ^2^Department of Emergency Medicine, the First Affiliated Hospital of Shenzhen University, Health Science Center, Shenzhen Second People's Hospital, Shenzhen, China; ^3^Shenzhen Hospital, University of Hong Kong, Shenzhen, China

**Keywords:** COVID-19, distress, healthcare provider, appraisal, coping

## Abstract

**Objective:** The central issue of this research is to evaluate the extent of cognitive appraisal and coping processes within the pandemic encounter and determines their influence on frontline healthcare providers who had been dispatched to the coronavirus disease 2019 (COVID-19) epicenter (HPDE) distress symptoms.

**Materials and methods:** An electronic survey of the HPDE and frontline healthcare providers who worked in their original medical facility (HPOF) was conducted from March 1 to 15, 2020. Two variables, appraisal (measured with an 18-item questionnaire) and coping (measured The Brief Cope questionnaire), were used in the analysis to explain distress symptoms (Impact of Event Scale-Revised).

**Results:** A total of 723 eligible respondents completed the survey with a response rate of 57.3% (351 HPDE and 372 HPOF). The mean IES-R scores of HPDE respondents were 26.47 ± 11.7. Of HPDE respondents, 246 (70.09%) reported distress symptoms (score 9–88). The scores of intrusion, avoidance, and hyperarousal for HPDE were 10.28 ± 4.7, 8.97 ± 4.3, and 7.20 ± 3.2, respectively. The respondents had higher scores in overall distress and three subscales than HPOF. Appraisal and coping variables explained 77% of the distress variance. Five appraisal variables (health of self, health of family/others, virus spread, vulnerability or loss of control, and general health) were positively associated with distress symptoms. Four coping variables (active coping, positive reframing, self-distraction, and behavioral disengagement) were negatively associated with distress level, whereas self-blame was positively associated with distress symptoms. Regarding the appraisal, the scores of HPDE were significantly higher than HPOF (all *p*-values < 0.05), whereas being isolated was not significantly different between HDPE nurses and HPOF nurses. HPDE was significantly more likely to use humor, emotional support, instrumental support, self-distractions, venting, substance use, denial, behavioral disengagement, and self-blame (*P* < 0.05), whereas HPOF was significantly more likely to use active coping and acceptance (*P* < 0.05). HPDE doctors were significantly more likely than nurses to use active coping and acceptance (*P* < 0.05), whereas HPDE nurses were significantly more likely to use emotional support and use self-blame (*P* < 0.05).

**Conclusion:** Frontline healthcare providers who had been dispatched to the COVID-19 epicenter respondents had a higher distress level. Therefore, we should provide proactive psychological support based on specific appraisal and coping variables.

## Introduction

Coronavirus disease 2019 (COVID-19) emerged in China in December 2019 and rapidly led to a significant global health crisis (Phelan et al., 2020). Globally, as of May 11, 2021, there have been over 5.5 million confirmed cases and over 90,000 deaths (WHO, 2021). Previous studies revealed a profound and wide range of psychological distress among healthcare workers during the 2003 SARS outbreak (Wang et al., 2005). The COVID-19 pandemic has also generated widespread public panic and psychological distress among the general population and medical staff (Holmes et al., 2020; Lai et al., 2020; Fukase et al., 2021). A recent study showed that COVID-19 confirmed patients had a 33.62% incidence of neurological or psychiatric sequelae in the following six months, in which 12.84% had received their first such diagnosis (Taquet et al., 2021). In addition, frontline healthcare staff exposed to COVID-19 were at higher risk of occupational stress and psychological symptoms (Manh Than et al., 2020; Feingold et al., 2021). Some reports revealed that the increasing number of COVID-19 patients and suspected cases, exhaustion, isolation, and lack of proper psychological support could increase the emotional burden and cause high levels of distress among health workers (Bao et al., 2020; Chew et al., 2020; Holmes et al., 2020; Wang et al., 2020).

Since January 23, 2020, many frontline healthcare providers who had been dispatched to the COVID-19 epicenter (HPDE) were redeployed to Wuhan and Hubei province, where the medical system was on edge because of the severe COVID-19 epidemic (Yang et al., 2021); however, research about distress among HPDE is still rarely reported. In addition, HPDE had to work in an unfamiliar environment far away from their families and original clinical facilities, which could increase their distress levels compared with those who worked in their original medical facilities. So, it is essential to compare HPDE and frontline healthcare providers who worked in their original medical facility (HPOF) when evaluating the psychological distress of HPDE. Until we fully understand HPDE distress symptoms within the context of the COVID-19 pandemic, accurate intervention for HPDE distress symptoms cannot be properly provided. This has become a matter of urgency, as many countries are suffering second or third waves of the COVID-19 epidemic, and much medical staff needs to be redeployed (Fukase et al., 2021).

The cognitive processes of health providers who were experiencing psychological distress caused by COVID-19 have led to the current situation of evaluation (appraisal) and management (coping) (Lazarus and Folkman, 1984). Appraisal and coping are critical pathways to mitigate HPDE distress levels based on the clinical psychological model of S. Folkman (Folkman et al., 1986). That said, what are the predictor variables of appraisal and coping that can affect HPDE distress levels and what are the different effects of these predictors on HPDE and HPOF? These questions remain uncertain. So, we aimed to evaluate the extent of cognitive appraisal and coping processes within the pandemic encounter and determine their influence on HPDE distress symptoms compared with HPOF.

## Materials and Methods

### Setting and Participants

The study was a cross-sectional survey using an anonymous online questionnaire “questionnaire star.” Questionnaire star is an online crowdsourcing platform of China, like Amazon Mechanical Turk (Wu et al., 2018). Questionnaires access were made using Q.R. codes, and then, it was circulated to all participants *via* WeChat accounts. The participants could fill and upload the questionnaire in the WeChat app. A contact person in each medical facility was responsible for the distribution of the questionnaires. Data were collected from May 1 to May 15, 2020. The inclusion criteria were those frontline medical providers involved in managing, transferring, and caring for COVID-19 patients and willing to participate in this study. The questionnaire for HPDE had to be finished in 1 week after they arrived at their destinations. The questionnaire was in Chinese, and the return was also anonymous.

This study was approved by the hospital ethics committee.

### Measure

The level of distress symptoms was measured by the Impact of Event Scale-Revised (IES-R; range, 0–88). The IES-R has been well-validated to assess the extent of psychological impact after exposure to stressful circumstances in the Chinese population (Zhang et al., 2014). Items are rated on a 5-point scale ranging from 0 (“not at all”) to 4 (“extremely”). The total scores of IES-R were interpreted as normal (0–8), mild (9–25), moderate (26–43), and severe (44–88), and subscale scores can also be calculated for the intrusion, avoidance, and hyperarousal subscales (Tan et al., 2021).

Appraisals were assessed by a 19-item questionnaire which proved to be validated in a previous study on the 2003 SARS outbreak among health care workers (Wang et al., 2005). A 4-point Likert-type scale was used to rate the compatibility of each item with a current appraisal of participants (0 = cannot completely describe my situation, 1 = cannot describe my situation, 2 = can describe my situation, and 3 = can completely describe my situation). All items were grouped into six subscales (the health of self, the health of family/others, virus spread, vulnerability/loss of control, changes in work, and general health). The score of each subscale was obtained by the mean of items scores of its subgroup. Thus, the subscale scores were identified as the current appraisal rate of participants for these six subscales.

Coping was measured using the Brief Cope questionnaire (Kato, 2015). All 28 coping methods of the questionnaire were grouped into 14 subscales (acceptance, active coping, positive reframing, planning, using emotional support, humor, using instrumental support, venting, self-distraction, religion, self-blame, denial, behavioral disengagement, and substance use). Each subscale owns two coping methods. How each item was adopted was rated by a 4-point Likert-type scale (1 = I have not been doing this at all, 2 = I have been doing this a little bit, 3 = I have been doing this a medium amount, and 4 = I have been doing this a lot). The score of each subscale was obtained by the mean of items scores of its subgroup. Thus, the subscale scores were identified as the rate of adoption of the participant for these 14 subscales.

### Statistical Analysis

Statistical analyses were performed using Excel (Microsoft, Redmond, WA, U.S.A.) and Spss 23.0. Descriptive data were tested by Chi-Square (χ^2^) test between groups. The reliability of the instruments of the study was evaluated using Cronbach's α coefficients. The normal distribution of the variables was tested by the Kolmogorov-Smirnov test. Normal measurement data were shown as mean and SD. The Student's *t*-test was used to determine whether the HPDE and HPDF or different occupations differed within each of the three sets of predictors (IES-R, appraisal, and coping). For qualitative data, the χ^2^ test was used to compare the grades of IES-R in response to HPDE and HPOF groups. Multivariable linear regression with IES-R score as a dependent variable evaluates the association between HPDE distress level with appraisal and coping variables after adjusting for confounders, including age, gender, marriage, and occupation. All the tests were two-tailed, with a significance of *P* < 0.05.

## Results

A total of 1,262 medical staff [631 (50.0%) HPDE and 631 (50.0%) HPOF] were invited to participate in this study. In the end, 723 eligible respondents completed the survey with a response rate of 62.3%. Of all respondents, 351 (48.55%) participants were HPDE staff, and 372 (51.45%) participants were HPOF staff. In addition, 449 (62.10%) were female and 274 (37.90%) were male. Most participants were aged between 30 and 45 years [397 (54.91%)] and were married with children [503 (69.57%)]. About 314 (43.43%) participants were nurses, and 409 (56.57%) participants were doctors. There was no significant difference in characteristics between the two groups (Table 1).

**Table 1:** Characteristics of respondents.

**Characteristics**	**HPDE** ***N* (%)**	**HPOF** **(*N* = 372)**	**χ** ^**2**^	**Overall** ***p*-value**
Sex	2.753	0.097		
Men	116 (33.05%)	145 (38.98%)		
Women	235 (62.95%)	227 (61.02%)		
Ages (years)	6.694	0.082		
Below 29	145 (41.31%)	150 (40.32)		
30–45	173 (49.29%)	166 (44.62)		
46–59	33 (9.40%)	54 (14.52)		
60 and above	0 (0%)	2 (0.54%)		
Years of service	5.417	0.067		
0–4	49 (13.96%)	51 (13.71%)		
5–9	112 (31.91%)	91 (24.66%)		
10 and over	190 (54.13%)	230 (61.83%)		
Marriage status	5.090	0.165		
Unmarried	42 (11.97%)	49 (13.17%)		
Married without Child	33 (9.40%)	19 (5.11%)		
Married with Children	272 (77.49%)	299 (80.38%)		
Divorced or be widowed	4 (1.14%)	5 (1.34%)		
Occupation			1.910	0.167
Doctor	165 (47.01%)	194 (52.15%)		
Nurse	186 (52.99%)	178 (47.85%)		

*HPDE, frontline healthcare providers who had been dispatched to the epicenter of coronavirus disease 2019 (COVID-19) in China; HPOF, frontline healthcare providers in the original medical facility*.

*P < 0.05 was considered statistically significant*.

The mean IES-R scores of HPDE respondents were 26.47 ± 11.7. Of HPDE respondents, 199 (56.70%) reported mild distress symptoms (score 9–25), 34 (9.69%) reported moderate distress symptoms (scores 26–43), and 13 (3.70%) reported severe distress symptoms (score 44–88). The scores of intrusion, avoidance, and hyperarousal for HPDE were 10.28 ± 4.7, 8.97 ± 4.3, and 7.20 ± 3.2, respectively. The HPDE had higher scores in overall distress and three subscales (intrusion, avoidance, and hyperarousal) than HPOF. The distress scores of nurses were significantly higher than the distress scores of doctors (*P* < 0.05). The nurse from HPDE had higher distress scores than the doctor from HPOF (*P* < 0.05). The effect size for overall distress and the three subscales between the HPDE and HPOF was 0.78, 0.65, 0.63, and 0.98 (Cohen's d) (Table 2).

**Table 2:** Impact Event Scale-Revised.

**Variable**	**HPDE**	**HPOF**	**Statistics**	**Effect size**	***p*-value**
IES-R	26.47 ± 11.7	18.14 ± 10.6	10.049	0.78^c^	0.00^*^
Normal	109	191	41.77^b^	0.24^d^	0.00^*^
Mild	155	128			
Moderate	34	32			
Severe	13	21			
Subgroup					
Intrusion	10.28 ± 4,7	7.37 ± 4.2	8.774^a^	0.65^c^	0.00^*^
Avoidance	8.97 ± 4.3	6.39 ± 3.9	8.456^a^	0.63^c^	0.00^*^
Hyperarousal	7.20 ± 3.2	4.38 ± 2.6	13.128^a^	0.98^c^	0.00^*^

*HPDE, frontline healthcare providers who had been dispatched to the epicenter of COVID-19 in China; HPOF, frontline healthcare providers in the original medical facility*.

a *χ^2^-value*.

b *t-value*.

c *Cohen's d*.

d *Cramer's V*.

* *P < 0.05 was considered statistically significant*.

The scores of HPDE were significantly higher than the scores of HPOF (all *p*-values < 0.05) regarding the appraisal. The effect size for all appraisal variables between HPDE and HPOF was larger than 0.5 except for being isolated (0.17) and general health (0.43). There was a significant difference in appraisal between HDPE nurses and HPOF nurses, except being isolated. The effect size of the health of self between different occupations of HPDE was larger than 0.2, the rest effect size of appraisal variables was under 0.2 (Table 3).

**Table 3:** Appraisal.

**Variable**	**Working Position**					**Occupation of HPDE**				
	**HPDE**	**HPOF**	**Cohen's d**	***t*-value**	***p*-value**	**Doctor**	**Nurse**	**Cohen's d**	***t*-value**	***p*-value**
Health of self	2.73 ± 0.5	2.26 ± 0.5	0.87	11.71	0.00^*^	2.64 ± 0.5	2.80 ± 0.5	0.22	−2.94	0.00^*^
Health of family/others	2.92 ± 0. 6	2.47 ± 0.6	0.79	10.67	0.00^*^	2.85 ± 0.6	2.99 ± 0.6	0.17	−2.34	0.00^*^
Virus spread	3.01 ± 0.6	2.54 ± 0.6	0.80	10.72	0.00^*^	2.93 ± 0.6	3.00 ± 0.5	0.18	−2.46	0.00^*^
Vulnerability orloss of control	2.64 ± 0.6	2.26 ± 0.6	0.63	8.48	0.00^*^	2.61 ± 0.5	2.67 ± 0.6	0.07	−0.92	0.00^*^
Changes in work	2.41 ± 0.6	1.98 ± 0.6	0.68	9.22	0.00^*^	2.30 ± 0.6	2.51 ± 0.7	0.25	−3.32	0.00^*^
Being isolated	2.30 ± 0.7	2.19 ± 0.7	0.17	2.34	0.02^*^	2.26 ± 0.7	2.34 ± 0.7	0.10	−1.30	0.19

*HPDE, frontline healthcare providers who had been dispatched to the epicenter of COVID-19 in China; HPOF, frontline healthcare providers in the original medical facility*.

*Scores ranged from 0 to 3 of the first six dimensions, the score of general health ranged from 1 to 4. Scores were shown as Mean ± SD*.

* *P < 0.05 was considered statistically significant. Reliabilities (Cronbach's α) for the above six dimensions were 0.83, 0.82, 0.83, 0.85, 0.83, and 0.85*.

Frontline healthcare providers who had been dispatched to the COVID-19 epicenter were significantly more likely to use humor, emotional support, instrumental support, self-distractions, venting, substance use, denial, behavioral disengagement, and self-blame (*P* < 0.05). Whereas, HPOF was significantly more likely to active coping and acceptance (*P* < 0.05). The effect size of self-distractions between different occupations of HPDE was larger than 0.5 (0.55), whereas active coping, humor, emotional support, instrumental support, venting, denial, behavioral disengagement, and self-blame were in the 0.2–0.5 and religion was under 0.2.

Frontline healthcare providers who had been dispatched to the COVID-19 epicenter doctors were significantly more likely than nurses to use active coping and planning (*P* < 0.05), whereas HPDE nurses were significantly more likely to use emotional support, venting, denial, and self-distractions (*P* < 0.05). The effect size of the above variables was 0.2–0.5 except venting (0.2) (Table 4).

**Table 4:** Coping.

**Variable**	**Working place**					**0ccupation Of HPDE**				
	**HPDE**	**HPOF**	**Cohen's d**	***t*- value**	***p*-value**	**Doctor**	**Nurse**	**Cohen's d**	***t*- value**	***p*-value**
Acceptance	3.49 ± 0.6	3.60 ± 0.5	0.19	−2.49	0.01^*^	3.57 ± 0.5	3.42 ± 0.6	0.17	2.28	0.23
Active coping	3.57 ± 0.6	3.71 ± 0.6	0.25	−3.31	0.00^*^	3.67 ± 0.6	3.48 ± 0.7	0.30	3.95	0.00^*^
Positive reframing	3.27 ± 0.7	3.32 ± 0.7	0.07	−0.88	0.38	3.29 ± 0.7	3.26 ± 0.7	0.02	−0.30	0.77
Planning	3.23 ± 0.7	3.30 ± 0.8	0.10	−1.40	0.16	3.35 ± 0.7	3.11 ± 0.77	0.32	4.36	0.00^*^
emotional support	1.35 ± 0.6	1.20 ± 0.5	0.32	4.29	0.00^*^	1.25 ± 0.6	1.44 ± 0.7	0.31	−4.15	0.00^*^
Humor	1.67 ± 0.74	1.43 ± 0.7	0.35	4.68	0.00^*^	1.74 ± 0.8	1.61 ± 0.7	0.12	1.55	0.12
instrumental support	2.44 ± 0.8	2.30 ± 0.8	0.17	2.33	0.02^*^	2.41 ± 0.8	2.48 ± 0.8	0.20	−2.70	0.01
Venting	2.25 ± 0.8	1.92 ± 0.7	0.44	5.90	0.00^*^	2.22 ± 0.8	2.28 ± 0.8	0.02	−0.32	0.00^*^
Self-distraction	2.87 ± 1.0	2.30 ± 1.1	0.55	7.45	0.00^*^	2.76 ± 1.0	2.97 ± 1.0	0.41	−5.49	0.00^*^
Religion	1.67 ± 0.6	1.58 ± 0.6	0.16	2.18	0.03^*^	1.67 ± 0.7	1.67 ± 0.6	0.13	−1.70	0.10
Self-blame	1.71 ± 0.7	1.57 ± 0.6	0.22	2.90	0.00^*^	1.61 ± 0.7	1.82 ± 0.8	0.14	1.86	0.06
Denial	2.43 ± 0.9	2.15 ± 0.9	0.32	4.29	0.00^*^	2.36 ± 1.0	2.48 ± 0.9	0.29	−3.95	0.00^*^
Behavioral disengagement	1.22 ± 0.5	1.11 ± 0.4	0.26	3.43	0.00^*^	1.18 ± 0.5	1.25 ± 0.5	0.13	−1.34	0.18
Substance use	1.29 ± 0.6	1.16 ± 0.4	0.25	3.35	0.00^*^	1.34 ± 0.7	1.26 ± 0.6	0.13	1.24	0.22

*HPDE, frontline healthcare providers who had been dispatched to the epicenter of COVID-19 in China; HPOF, frontline healthcare providers in the original medical facility*.

*Scores ranged from 1 to 4. Scores were shown as Mean ± SD*.

* *P < 0.05 was considered statistically significant*.

*Reliabilities (Cronbach's α) for the above 14 dimensions were (0.73, 0.73, 0.73, 0.73, 0.70, 0.73,0.69,0.70,0.72,0.72,0.72,0.73,0.73, and 0.73)*.

The health of self, the health of family/others, and virus spread were positively associated with HPDE level, whereas the health of self, virus spread, and being isolated were positively associated with HPOF distress level. Three coping variables (active coping, positive reframing, and emotional support) were negatively associated with the HPDE distress level, whereas only active coping was negatively associated with HPOF distress. Five coping variables (acceptance, venting, self-blame, denial, and substance abuse) were positively associated with the HPDE distress level, whereas acceptance, venting, and denial were positively associated with the HPOF distress level (Table 5).

**Table 5:** Association between IES-R scores with appraisal and coping variables, in multivariable linear regression analysis with IES-R as dependent variable (*N* = 723).

**Adjust**	**HPDE**		**HPOF**	
**Predictor variable**	***β*** **(95%CI)**	***p-*value**	***β*** **(95%CI)**	***p-*value**
**Appraisal**				
Health of self	1.82 (0.26, 3.37)	0.02^*^	1.20 (−0.11, 2.51)	0.07
Health of family/others	2.43 (0.48, 4.38)	0.02^*^	1.58 (−0.39, 3.56)	0.12
Virus spread	3.92 (1.49, 6.34)	0.00^*^	4.85 (1.70, 8.00)	0.00^*^
Vulnerability or loss of control	0.28 (−1.49, 2.06)	0.76	0.24 (−1.24, 1.73)	0.75
Changes in work	1.15 (−0.08, 2.39)	0.07	1.00 (−0.06, 2.06)	0.07
Being isolated	0.73 (−1.72, 3.19)	0.56	2.65 (0.34, 4.95)	0.02^*^
**Coping**				
Acceptance	2.13 (0.04, 4.22)	0.04^*^	1.63 (0.39, 2.87)	0.01^*^
Active coping	−2.28 (−3.63, −0.93)	0.00^*^	−1.58 (−3.08, −0.08)	0.04^*^
Positive reframing	−2.08 (−3.45, −0.71)	0.00^*^	−1.49 (−3.00, 0.02)	0.05
Planning	0.28 (−1.49, 2.06)	0.76	0.24 (−1.24, 1.73)	0.75
emotional support	−1.16 (−2.27, −0.05)	0.04^*^	−0.07 (−2.10, 1.95)	0.94
Humor	0.28 (−1.64, 2.20)	0.77	−0.02 (−1.62, 1.58)	0.98
instrumental support	1.23 (−1.60, 4.07)	0.39	0.05 (−1.98, 2.09)	0.96
Venting	3.55 (2.06, 5.04)	0.00^*^	2.39 (0.87, 3.92)	0.00^*^
Self-distraction	−0.71 (−2.77, 1.35)	0.50	1.24 (−0.64, 3.11)	0.20
Religion	0.85 (−1.10, 2.80)	0.40	−0.66 (−3.19, 1.88)	0.61
Self-blame	2.97 (1.26, 4.68)	0.00^*^	1.66 (−0.13, 3.46)	0.07
Denial	2.24 (0.25, 4.23)	0.03^*^	2.20 (−0.09, 4.50)	0.06
Behavioral disengagement	−0.18 (−2.36, 2.00)	0.87	0.86 (−0.97, 2.69)	0.36
Substance use	2.43 (0.48, 4.38)	0.02^*^	1.58 (−0.39, 3.56)	0.12

*HDPE, frontline healthcare providers who had been dispatched to the epicenter of COVID-19 in China; β, standardized β coefficient; CI, confidence interval*.

*Adjust the model for age, sex, marriage, and occupation*.

* *P < 0.05 was considered statistically significant*.

## Discussion

In this study, nearly 60% of participants experienced psychological distress. We found that HPDE participants suffered more distress symptoms than HPOF. The result was consistent with one previous study (Lai et al., 2020). This study found that Wuhan and Hubei province healthcare workers were at especially higher risk for distress symptoms compared with others; however, the studies of health providers in this research did not consist only of HPDE but also those whose original working facilities were located in Wuhan and Hubei province. As they were working in an unfamiliar environment and far away from their families and original clinical facilities, HPDE had to face the stress of local health workers, an unfamiliar medical specialty, and being away from their families. We infer that the severity of HPDE distress symptoms was neglected before and was also underestimated. Furthermore, the magnitude of the effects of the HPDE on three subscales was not the same. The hyperarousal effect was greater than the other two variables, possibly because HPDE also lacked sufficient knowledge about the virus at the beginning of the pandemic, especially when entering unfamiliar environments. That is why many HPDE suffered more hyperarousal symptoms.

Studies have shown that job-related distress of healthcare workers was mainly associated with their health and the fear of infecting their families, social isolation, uncertainty, reluctance to work, and other appraisals (Barello et al., 2020). In this study, with regards to their distress symptoms, HPDE was mainly concerned with the health of self and family/others and virus spread, whereas health of self, virus spread, and being isolated was the concerns of HPOF. These results were consistent with the previous study, revealing that health and safety were the main concerns of the staff among the various appraisals related to the epidemic outbreak (Khalid et al., 2016). There were greater concerns of all six appraisals among HPDE than HPOF, which was also evident for worse distress symptoms of HPDE.

However, HPDE was more concerned about the health of family/others than being isolated, whereas HPOF was more concerned about being isolated (Table 5). The underlying cause may be that while providing medical assistance in Hubei province, most healthcare HPDE stayed together when working or resting; however, they also had no contact with their families. This feature of HPDE could decrease the worry of being an isolated factor and increase families/others. HPDE nurses were more worried in all six appraisals than doctors in the HPDE subgroup, indicating that nurses were suffering more distress than doctors (Folkman, 1986; Mosheva et al., 2020). This was also consistent with the IES-R scores. Therefore, more assistance should be provided to HPDE nurses to alleviate their distress symptoms.

Overall, coping might adversely affect distress symptoms (Mosheva et al., 2020); however, not all the coping variables are negatively correlated with HPDE distress. Previous studies revealed that planful problem-solving coping was negatively correlated with symptoms, whereas confrontive coping was positively correlated (Folkman, 1986). In this study, HPDE adopted more planful problem-solving copings and less confrontive coping than HPOF (Table 4). HPDE was supposed to have fewer distress symptoms compared with HPOF based on the above coping theory; however, HPDE had higher distress scores in this study. The reason is that HPDE encountered more stress and lacked sufficient approaches to problem-solving coping. Active coping and planning were adopted more by HPDE doctors. On the contrary, self-blame, venting, denial, and emotional were more adopted by HPDE nurses, which revealed that HPDE nurses used more confrontive coping than HPDE doctors and consequently had higher distress levels. The cause might be that the duties of the doctors were to provide treatment based on updated medical information, so they had better access to the latest COVID-19 information.

Among the coping strategies, positive reframing, emotional support, self-blame, and substance abuse could influence HDPE distress symptoms positively or negatively, whereas active coping, acceptance, and venting could influence both HDPE and HPOF. A possible interpretation of this finding is that HPDE needs more assistance to relieve their distress symptoms, and such assistance would be of more benefit to their distress symptoms as the *β* value of the above coping variables was larger in HPDE groups than in the HPOF groups. Thus, theoretically, more distress symptoms will be relieved in HPDE when one specific coping strategy is improved.

Many studies have reported that positive coping or other practices could relieve the psychological impact (Zaçe et al., 2021). The same finding was noticed in this study, improving active coping skills and other planful problem-solving coping support measures and decreasing the self-blame influence of distress symptoms on HPDE will be useful; however, HPDE nurses need more support to manage these confrontive coping influences.

This study has several strengths. First, it is a comprehensive study of cognitive appraisal and the coping processes encountered during the pandemic, and it analyzes their influence on distress symptoms among HPDE, who have been mostly neglected. Second, the survey began during the peak of the COVID-19 outbreak in China. So, the timing of this study allowed healthcare providers to describe their acute distress symptoms and current appraisal and coping mechanisms; however, this study still has several limitations. First, the surveys for the HPDE and HPOF groups were not conducted at the same time. Therefore, the impact of COVID-19 on each group may differ. Second, the questionnaire used to measure the appraisal had not been fully validated, as it was only used in the SARS epidemic in 2003. Finally, there could be a potential reporting bias since medical staff might under-report their distress levels during the global pandemic.

## Conclusion

We believe that COVID-19 provoked a high level of distress among HPDE. Furthermore, the relations between appraisal variables and planful problem-solving coping were positively correlated with distress levels among HPDE, whereas confrontive coping was negatively correlated. Therefore, we should plan ahead of a medical assistance mission to provide proactive psychological support to frontline medical staff, based on the nature of the mission and specific appraisal and coping variables.

## Data Availability Statement

The raw data supporting the conclusions of this article will be made available by the authors, without undue reservation.

## Ethics Statement

The studies involving human participants were reviewed and approved by Shenzhen University General Hospital. The patients/participants provided their written informed consent to participate in this study.

## Author Contributions

ZJ and KL drafted the manuscript. ZJ collected the epidemiological and clinical data. KL is responsible for summarizing all clinical data. ZD and WH revised the final manuscript. All authors contributed to the article and approved the submitted version.

## Conflict of Interest

The authors declare that the research was conducted in the absence of any commercial or financial relationships that could be construed as a potential conflict of interest.

## Publisher's Note

All claims expressed in this article are solely those of the authors and do not necessarily represent those of their affiliated organizations, or those of the publisher, the editors and the reviewers. Any product that may be evaluated in this article, or claim that may be made by its manufacturer, is not guaranteed or endorsed by the publisher.
